# Supplementary material for: Serum Extracellular Vesicle-Derived hsa-miR-2277-3p and hsa-miR-6813-3p Are Potential Biomarkers for Major Depression: A Preliminary Study
Source: Int J Mol Sci. 2023 Sep 9;24(18):13902. doi: 10.3390/ijms241813902 (PMC10531403; doi:10.3390/ijms241813902)
Supplement: Supplementary file 1 [file ijms-24-13902-s001.zip › tableS1.pdf]

Supplementary Table 1. Differential Expression of miRNAs in MD Cases: Significant Changes in 28 miRNAs.

Factor	Severity			*p*.value
	Mild	Moderrate	Severe	
number	3	10	3	
hsa-let-7f-1-3p	11.98	9.082	7.194	3.657e-2*
hsa-miR-1236-5p	16.86	33.27	12.75	2.839 e-2
hsa-miR-1306-5p	7.854	17.70	0	3.647 e-2
hsa-miR-150-5p	14.43	4.636	12.51	4.541 e-2
hsa-miR-191-3p	15.57	6.156	12.71	4.12 e-2
hsa-miR-1910-5p	0	14.12	0	2.945 e-2
hsa-miR-2277-3p	7.750	6.410	0	3.423 e-2*
hsa-miR-2392	26.59	143. 0	37.41	3.267 e-2
hsa-miR-25-5p	17.35	38.36	0	2.575 e-2
hsa-miR-3177-5p	19.31	13.93	19.84	3.298 e-2
hsa-miR-3190-3p	25.86	85.23	27.10	4.307 e-2
hsa-miR-365a-5p	41.97	152.2	29.69	2.82 e-2
hsa-miR-4436b-5p	0	19.46	0	3.813 e-2
hsa-miR-4646-5p	13.57	58.25	0	2.805 e-2
hsa-miR-4681	0	16.32	0	3.347 e-2
hsa-miR-4721	0	52.38	0	3.716 e-2
hsa-miR-4726-3p	0	7.599	0	3.158 e-2
hsa-miR-483-3p	0	6.129	0	3.158 e-2
hsa-miR-483-5p	0	34.88	0	3.991 e-2
hsa-miR-490-3p	16.42	4.515	8.662	4.824 e-2
hsa-miR-516a-5p	17.47	6.039	17.25	3.716 e-2
hsa-miR-6070	18.66	5.945	15.88	4.187 e-2
hsa-miR-642a-5p	0	6.233	0	3.158 e-2
hsa-miR-6780b-3p	0	8.443	0	3.482 e-2
hsa-miR-6813-3p	23.40	15.49	11.23	2.748 e-2*
hsa-miR-6834-3p	0	7.996	0	3.482 e-2
hsa-miR-7113-3p	0	20.92	0	4.631 e-2
hsa-miR-718	18.86	13.29	19.13	6.224 e-3

*p<0.05 and decreasing expression with severity

Values in Table 3 represent miRNA signal values adjusted by subtracting the mean of the Negative Control ± 2SD. MiRNAs with non-positive adjusted values are shown as "0". Valid miRNAs have their adjusted signal values displayed.
